# Experimental Modeling of a Formula Student Carbon Composite Nose Cone

**DOI:** 10.3390/ma10060620

**Published:** 2017-06-06

**Authors:** Neil A. Fellows

**Affiliations:** Department of Mechanical Engineering and Mathematical Sciences, Faculty of Design, Technology and the Environment, Oxford Brookes University, Wheatley Campus, Wheatley, Oxford OX33 1HX, UK; neil.fellows@brookes.ac.uk

**Keywords:** CFRP, carbon composite, nose cone, Formual Student, numerical modeling

## Abstract

A numerical impact study is presented on a Formula Student (FS) racing car carbon composite nose cone. The effect of material model and model parameter selection on the numerical deceleration curves is discussed in light of the experimental deceleration data. The models show reasonable correlation in terms of the shape of the deceleration-displacement curves but do not match the peak deceleration values with errors greater that 30%.

## 1. Introduction

In order to win the Fédération Internationale de l’Automobile (FIA) Formula One (F1) series, racing teams compete to obtain the best racing drivers and produce the best racing cars, within their budgets and within the regulations set down by the sport’s governing body (FIA). An important area of racing car design, in terms of performance and safety, is the front nose cone. Its key function is to absorb energy during a frontal impact to reduce the duration and magnitude of the peak decelerations, as they are known to have a huge influence on driver survivability during impact events [1,2]. To ensure that the nose cone performs adequately, the FIA have set some minimum requirements, in terms of energy absorbed and maximum deceleration, which the nose cone must meet during impact testing [3,4]. To reduce the amount of tests required, F1 teams use numerical modeling to predict the crash performance [5]. Currently F1 nose cones are manufactured using carbon fiber reinforced composites (CFRP). Due to the commercial value of nose cone test data there is little published information available and what has been published is limited in terms of the properties and layup of the CFRP material and the modeling parameters used [5].

Formula Student is a competition that started regularly in the USA in 1981 and since 1998 has been held in the UK sponsored by the Institute of Mechanical Engineers (IMechE). The competitions are open to student teams from around the world to design, build, test and race a car [6]. The tight test circuits lend themselves to short wheel base vehicles and there are regulations governing things like safety features and engine capacity. The regulations are though a lot less restrictive than FIA F1 regulations particularly in terms of materials that can be used [4]. This gives a lot of scope to be innovative in terms of component design. Also as the competition is less commercially sensitive it is possible for more detailed publication of test and numerical data to be made available. Even so there is limited published work on modeling Formula Student composite nose cones. There are some publications using foam [7], aluminum honeycomb filled tubes [8,9] and metal frustum [10] as impact attenuators. There is also a paper that models CFRP nose cones, but only compares the results against static tests [11].

A key aspect in terms of modeling of vehicle impact is the determination of the decelerations generated during the impact event. The implemented failure and damage models will have a significant effect on the decelerations predicted. Stress-based progressive (or continuum) damage mechanic models, as used in this work, have failure criteria that try to capture the different failure modes of the composite such as fiber fracture, matrix cracking, fiber pull out, etc. [12,13]. They are relatively easy to implement in numerical codes, can discriminate between failure modes, can be implemented with limited material data and have been validated against many different problems. There is though limited validation of the available models for crash of composite structures. Also, when modeling, impact additional parameters are often introduced, such as the crush front softening parameter, that add extra non-physically defined variables [14]. In addition to the failure criteria post-failure damage degradation rules are used. They often do not degrade the material properties to zero to avoid numerical instabilities and also sometimes allow for a gradual degradation to account for post-failure strength of the material [15]. Degradation models are controversial in that they have limited physical rationale and are often not robust when applied to different problems. The work that has been carried out on modeling crashes of composites has shown that there are issues [10] but through calibration of material model parameters a predictive (although not physically based) model can be produced [14,16,17].

This paper therefore compares numerical models to experimental data obtained from an impact test carried on a CFRP nose cone to meet the regulations for the UK Formula Student 2012 competition [18]. To pass the regulations the average raw acceleration and the peak SAE 60 acceleration must be below set values. The numerical package used in this work was LS-Dyna (v6.1), which has been developed by Lawrence Software Technology Corporation for solving non-linear transient events [19]. The nose cone presented is a carbon composite nose cone, used on the Oxford Brookes XII Formual Student car. The effect of material models and modeling parameters are discussed in terms of the deceleration accuracy obtained in comparison to the test data.

## 2. Modeling Procedure

### 2.1. Geometry

**Figure 1:**
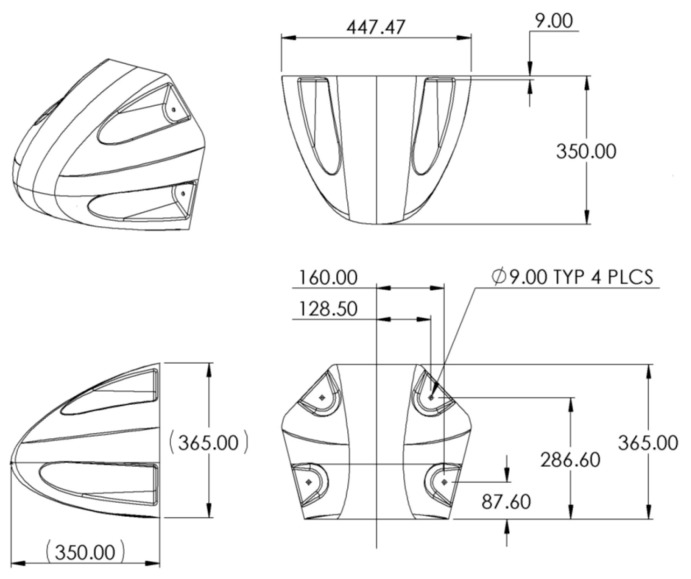
Geometry of Brookes XII carbon fiber reinforced composite (CFRP) nose cone [18].

The geometry of the nose and the dimensions are shown in Figure 1.

### 2.2. Mesh Generation

**Figure 2:**
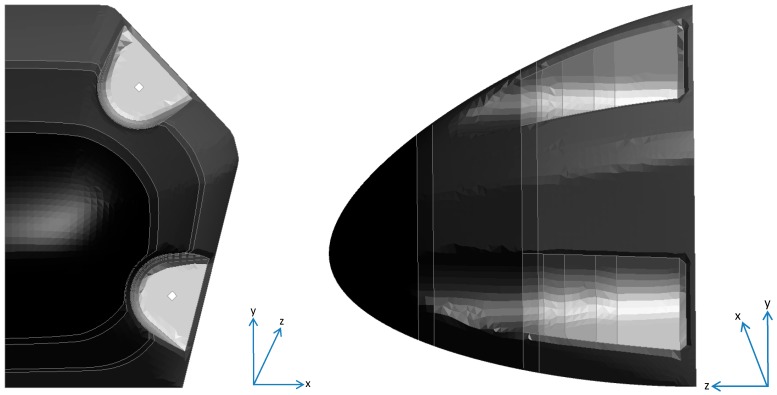
Boundary conditions applied in CFRP nose cone model.

The original drawing for the nose cone was developed in 3D using Solidworks. Primer, the pre-processor used in this work, requires a mesh of the geometry in order to set up the other model parameters. To create a mesh the outside top surfaces of Solidworks drawings were exported as a Step file into Catia. The model was then reduced in half by removing the surfaces on one side of the central symmetry line. This enables a symmetry boundary condition to be implemented, which improves computational speed. The surfaces were then joined and modified to have split lines at positions where there was a variation in laminar thickness (see Figure 2). 

Each split surface was then meshed using linear quad elements, with automatic mesh capture set to on, as shown in Table 1. This ensures that a continuous mesh is generated, while providing the ability to apply different laminar layups to each split surface. This reduces the need to produce contacts in the model between the split surfaces, increasing computational efficiency. The mesh was exported as a Nastran files (dat) for importing into OASYS Primer v13.0 [20]. OASYS Primer was used to set up the model parameters, such as boundary conditions, material properties, contact friction, impact velocity, etc., as discussed in Section 2.3 and Section 2.4. The shell elements used were the LS-Dyna default Belytschko-Lin-Tsay element [21] and the models were run with double precision.

### 2.3. Boundary Conditions

**Figure 3:**
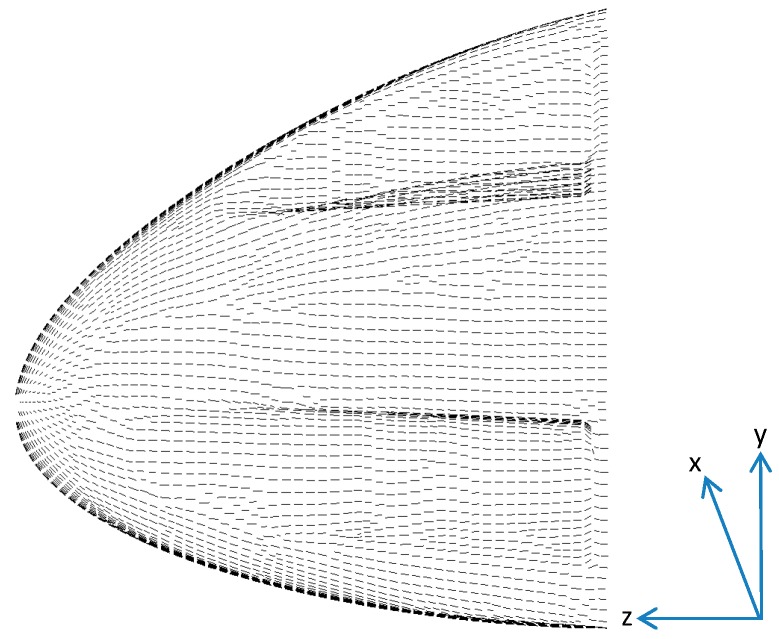
Orientation of composite material (line direction represents reference orientation).

The orientation of the composite material (Beta angle within LS-Dyna) was set so that the reference direction of the fabric material was aligned along the direction of impact (z direction), as shown by the direction of the lines in Figure 3. Each lamina within the composite is then normally given an additional angle to represent the orientation of the lamina with respect to the reference direction. Additional lamina orientations were not applied, due to the modulus value not varying with in-plane direction, as discussed in Section 2.4.

The impact was modeled by restraining the nose cone and using a rigid moving wall. The moving wall was given a mass of 150.77 kg (to account for the sled weight and also the weight of the anti-intrusion plate and mountings) and a velocity of 7.11 m/s. The mass was reduced in half from that in the test to account for halving the nose cone model by utilizing a symmetry boundary condition.

### 2.4. Material Properties and Layup

**Figure 4:**
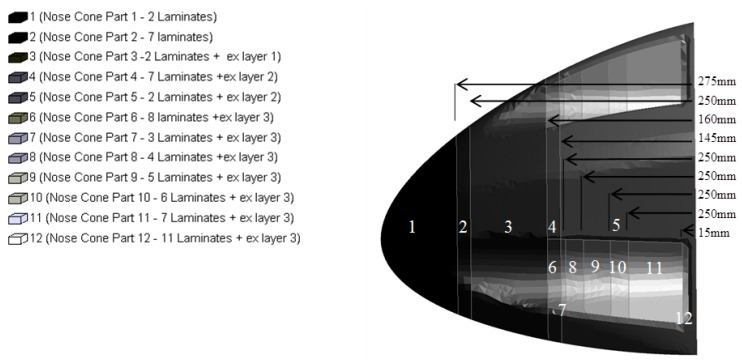
Composite layup of nose cone.

The material was laid up so that fibers were aligned orthogonally along and perpendicularly to the impact direction. Two global plies were applied across the whole nose cone and along the cut out sections extra layers were applied that were placed in between the global outer layer and global inner layer. Also, two additional 15 mm wide ribs of 5 plies were applied around the inner perimeter at 145 mm and 250 mm from the base on the nose cone as shown in Figure 4. Due to difficulty of accurately laying up exactly to the design drawing there was some extra fabric in the global layers. In the model each layer was represented by an integration point within the shell element. The amount of integration points across the shell thickness therefore varied from between 3 and 11.

The CFRP material used in the CFRP nose cone was a 2/2 twill weave MTM57/CF3202 prepreg with a cloth weight of 245 g/m^2^. The volume percentage of resin has been assumed to be 42% in line with MTM57/CF3200 [22] giving a prepreg weight of 422.4 g/m^2^. The cured thickness of the prepreg was 0.3 mm giving a density of 1408 kg/m^3^. The model mass using this density (with dimensions as shown in Figure 1) was 0.63 kg. To match the mass of the nose cone used in the test and to allow for material run outs (thickness measurements were taken from manufactured cone) part 3 was given an additional 0.164 mm layer, parts 4 to 5 were given additional 0.18 mm layers and parts 6 to 12 were given additional 0.24 mm layers. This gave a final model mass of 0.724 kg.

The main mechanical properties were obtained from Bartolotta et al. [23] and those that were not available were assumed to be the same as for CFRP CFS003/LTM25 (a similar 2/2 twill weave prepreg [24]) with properties obtained from Crews et al. [25], as shown in Table 2. LS-Dyna uses 11, 22 and 33 indices to represent the orthogonal directions of the composite material normal properties and 12, 13, 23, 21, 31 and 32 to represent the shear property directions. The 11 and 22 indices represent two in plane directions and the 33 index represents the perpendicular through thickness direction. For unidirectional materials, the material properties along the 11 direction are normally aligned with the fiber direction. For twill materials, as used in this work, the in-plane rotation of the material should not theoretically affect the Young’s modulus values and the Young’s modulus in the 11 and 22 directions were given the same value. In addition, the material model within LS-Dyna assumes that the Young’s modulus does not vary due to the sign of the loading and so only one value was entered for the 11 and 22 directions. All geometry, velocities, masses and material properties within the models were set to SI units (m-s-kg).

LS-Dyna has several stress-based composite CDM material failure models implemented as standard in the software package. The two main models are material model 54 [13], which is suitable for unidirectional composites, and material model 58 [14], which is appropriate for woven fabric composites. A key difference between the models is that material model 58 has identical failure criteria along the longitudinal and transverse directions whereas material 54 has different failure criteria in the two directions to account for the effect of strength and failure mode changes due to the fiber orientation. In this case, as the composite is a 2/2 twill weave material, model 58 has been used, with all the strengths input as positive values [26]. Details of the failure criteria used in material model 58 are given in the LS-Dyna manual [13] and in the work by Chatia [15]. Material 58 implements a damage evolution variable into the Hashin failure criteria [13,27,28]. In addition to the material properties; extra model parameters can be set that influence the material failure behavior of the model. Table 3 shows the additional material properties that were varied to investigate their effect on the deceleration of the rigidwall within the model.

If the failure surface is set to 1.0 the failure criteria in the longitudinal and transverse directions include a shear stress term as well as a tensile stress term. If the failure surface is set to −1.0 the shear stress is treated separately which means that shear damage parameter only modifies the shear stiffness and not the normal stiffnesses. The implementation of the damage parameters into the Hashin criteria changes the loading and post-failure stress strain behavior of the composite. Three damage parameters are incorporated, which are related to tension failure in the longitudinal and transverse directions, compression failure in the longitudinal and transverse directions and shear failure. The form of the damage variable is given in Equation (1) (defined in this case in terms of normal stress),

(1) ω=1−exp(1mexp(1)(εuts(σutsE))m)

where *σ_uts_*, *ε_uts_* and *E* are the ultimate tensile stress, ultimate tensile strain and Young’s modulus respectively. The variable m is given by Equation (2).

(2) m=1ln(εuts(σutsE))

**Figure 5:**
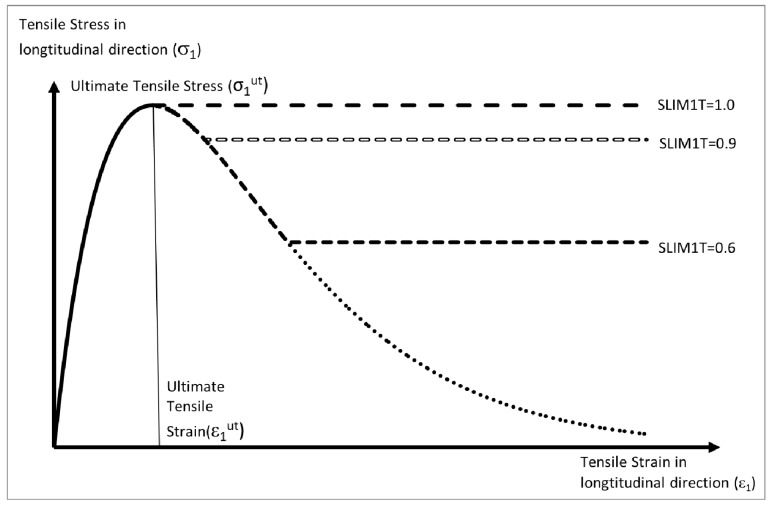
Effect of Slim factor (shown for longitudinal direction).

In Figure 5 the effect that the damage parameter has on the stress strain curve, along the longitudinal direction, when the laminate is loaded in tension is shown. Similar curves can be developed for the transverse direction and for compression loading. The curves can be further modified through the use of Slim factors that prevent the stresses falling below given limits after failure. The effect that different Slim1T values have is shown in Figure 5 (for example a Slim1T value of 0.6 will stop the stress dropping below 60% of the ultimate tensile stress). It is possible when using a faceted failure surface (FS = −1) to input a two-stage shear strain loading curve. The Slim S parameter can also be used to ensure the shear stress will not drop below a set value in a similar way to the normal stresses. After failure the stresses remain at the levels determined by the Slim factors until either the maximum effective strain (Erods) is reached or distortion within the element causes the time step to go below the time-step size (TSize). If either of these conditions occurs the element is deleted.

**Figure 6:**
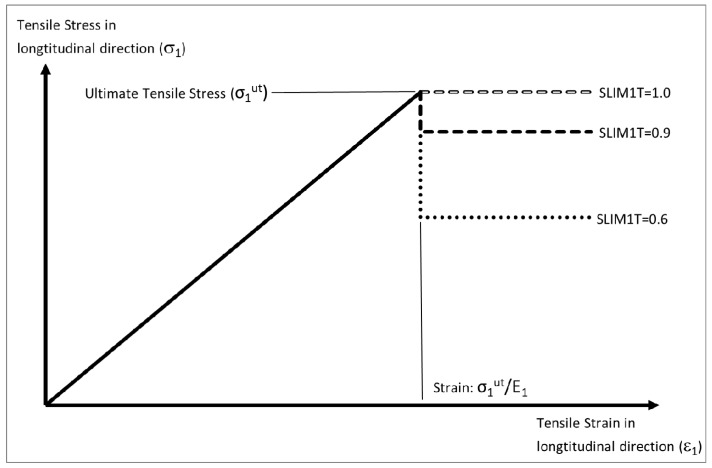
Effect of Slim factor (shown for longitudinal direction).

Although it is possible to incorporate non-linear loading within material 58 by inputting the ultimate strain values, as shown in Figure 5, this was not done in this work. Without ultimate strain values the stress strain behavior for both normal and shear stresses follows the behavior shown in Figure 6. The initial stress-strain curve is linear prior to reaching the ultimate tensile stress where immediate damage occurs, with the stress dropping down to the stress level defined by the Slim factor. This material stress-strain behavior is similar to that of material model 54. A key reason to prevent the material stress dropping to zero after failure is to prevent instability within the model. It is recommended for compressive loading that the Slim1C and Slim2C parameters are set to 1.0 [19].

The Soft factor in the material model reduces the stiffness of elements behind the crash front to enable gradual loading of the elements to ensure numerical stability. All other parameters were set to the default parameters apart from those shown in Table 4 and Table 5. The additional parameters in Table 5 were used in a few models to check their effect, over the default values, as discussed in the results section. The contact friction between the composite and the rigidwall and between composite and composite during failure were set as given in Table 6.

To study the various model parameters that influence post-failure material behavior several different sets of model runs were carried out as shown in Table 7, Table 8 and Table 9.

## 3. Experimental Testing

**Figure 7:**
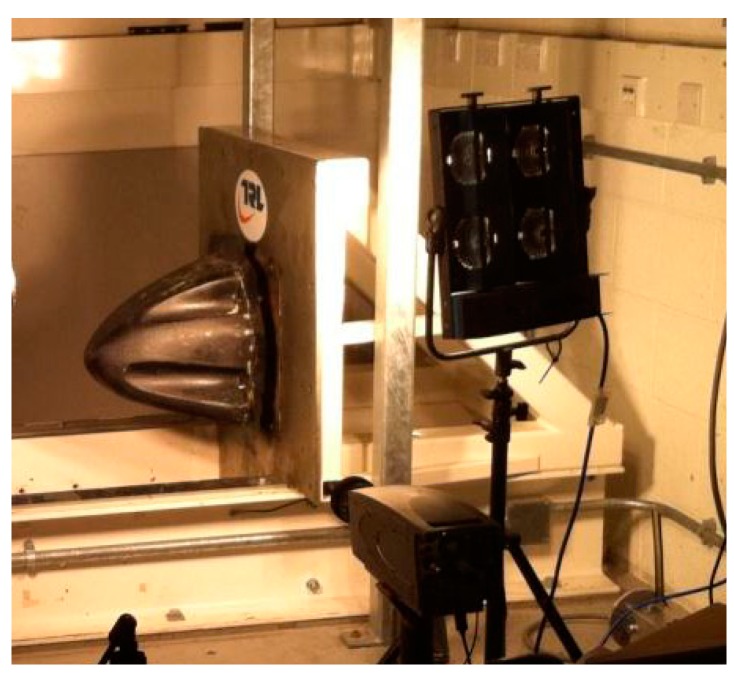
Sled impact tester at TRL [18].

The experimental test work was carried out at the Transport Research Laboratory (TRL) using a Sled Impact tester (see Figure 7).

**Figure 8:**
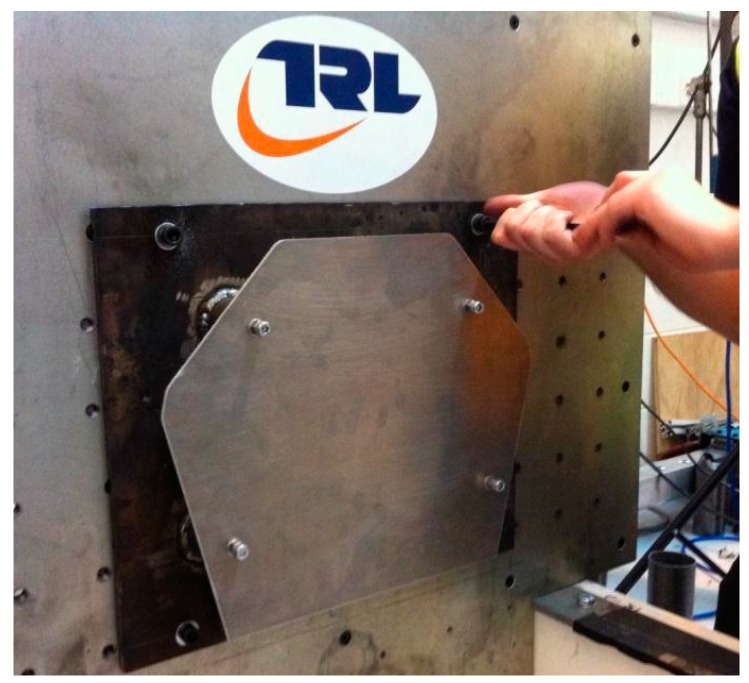
Nose cone mounting plate—showing 50 mm offset [18].

The nominal impact velocity was set to 7 m/s with a sled weight of 300 kg. The nose cone weight was 0.726 kg and the weight of the bolts and anti-extrusion plate was 1.543 kg. The velocity was measured using an infra detector operating at a frequency of 50 Hz [18]. The data logger was set to capture data at a frequency of 20 kHz. An SAE class 60 filter was applied to the raw displacement and deceleration test data, after the test, using the Oasys T/HIS data package. The nose cone mounting plate (anti intrusion plate) was offset from the sled impact face by 50 mm using four 8 mm Grade 8.8 bolts (see Figure 8).

**Figure 9:**
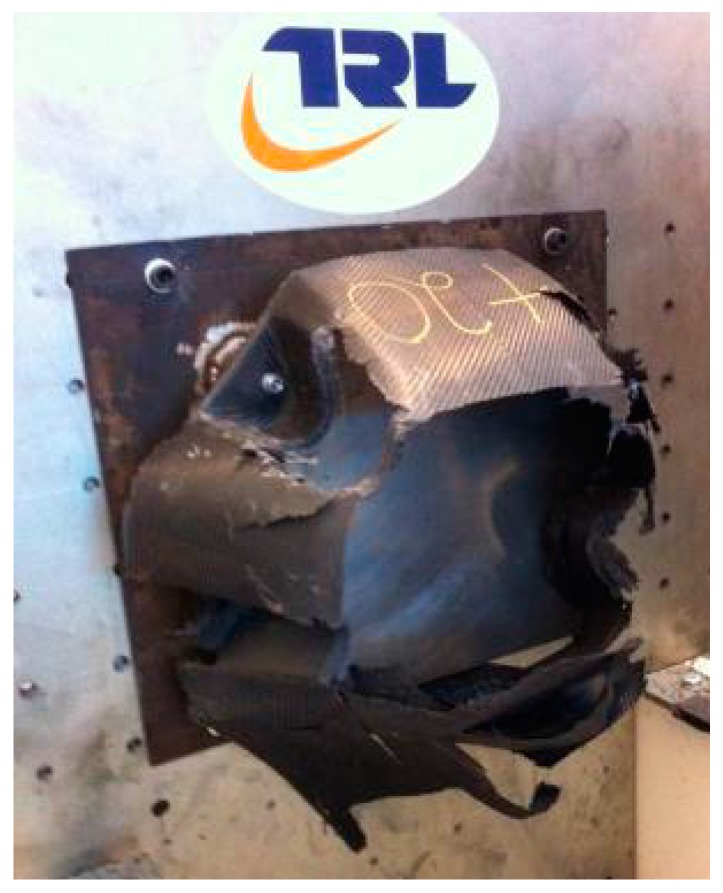
Carbon fiber nose cone after impact [18].

**Figure 10:**
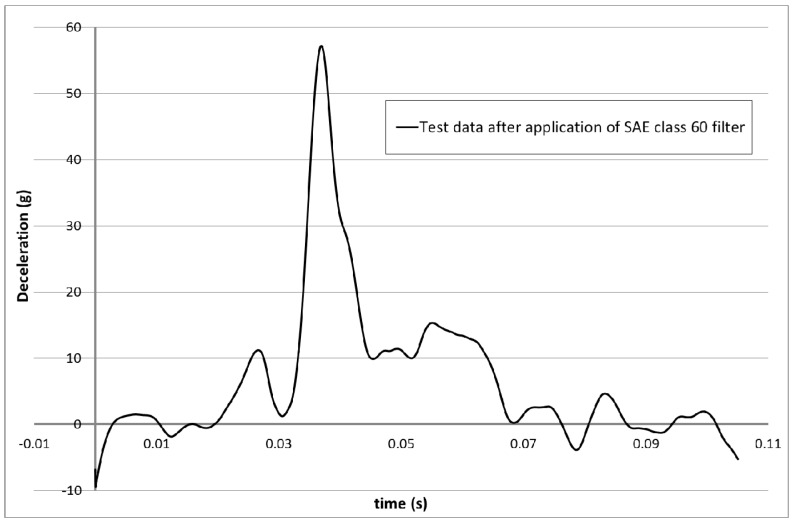
Deceleration against time obtained from the impact test.

The resultant damage to the nose cone and the deceleration against time data (SAE class 60 filtered) from the impact test is shown in Figure 9 and Figure 10.

## 4. Numerical Results

**Figure 11:**
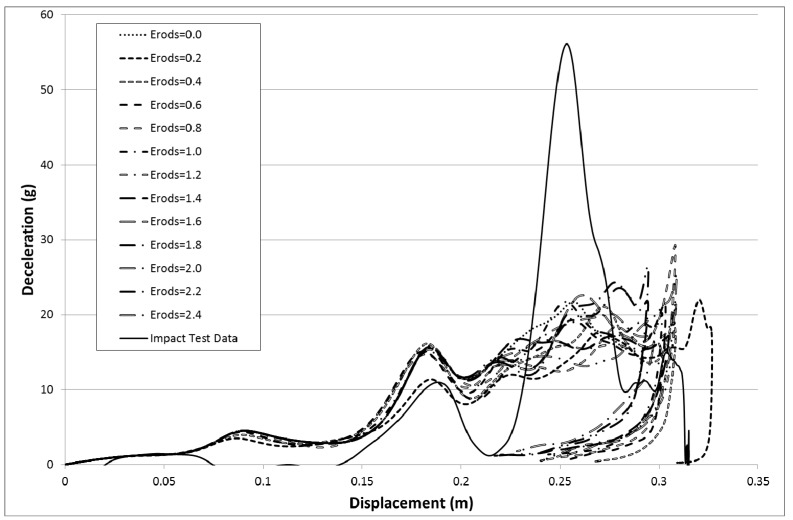
Effect of Erods parameter with All Slim = 1.0, TSize = 1 × 10^−11^, Soft = 1.0 and FS = 1.0.

To study the various material parameters the model runs shown in Table 7, Table 8 and Table 9 were carried out. The deceleration versus displacement results for the rigidwall within the model for various values of maximum effective strain (Erods) are shown in Figure 11. The model deceleration-time and displacement-time curves were imported into the Oasys T/HIS data package and a SAE class 60 filter applied before exporting them into Excel for plotting against each other. The model parameters were set as follows; element deletion would occur if the step size fell below 1 × 10^−11^ s (TSize = 1 × 10^−11^), element stresses would stay at the failure stress values after element failure (All Slim = 1.0), no softening would occur in front of the crash front (Soft = 1.0) and failure would be governed by a smooth failure surface (FS = 1.0). The deceleration and displacement of the rigidwall are plotted as positive values in all the figures in this paper. The figures have also been cropped, to focus only on deceleration, to increase the image size. This removes small sections of the experimental curve which showed low acceleration values.

Due to the number of curves generated it was decided that a clearer way to assess them against the experimental data was to find the maximum deceleration for each curve between the displacements of 0.23 m and 0.29 m and plot the results against each other as surface plots (see Figure 12, Figures 14–21 and Figures 24–26). Figure 12 shows the effect of changing the Erods parameter and the Soft factor while keeping all the other model values the same as for Figure 11 (Table 6—Set 1).

The results in Figure 13 are based on the same model parameters as used in Figure 11 but this time the failure surface (FS) was set to −1. The results for the facetted failure surface (FS = −1) were much closer to the experimental peak deceleration so all the proceeding models presented (Figure 13 onward) are based on a facetted failure surface (FS = −1) apart from data given later in Table 10 and Table 11 that examines run to run variation. Although the result for an Erods parameter of 0.4 gave the highest peak the highest peak that matches the position of the experimental peak was for an Erods parameter of 0.6.

Figure 14 shows the effect of changing the Erods parameter and the Soft factor while keeping all the other model values the same as for Figure 13 (Table 6—Set 2). The Erods and Soft factor can be seen to affect the results with the maximum peak at an Erods parameter of 0.2 and a Soft factor of 1.0. 

To examine the effect of Tsize the models that generated the results in Figure 14 were repeated but using a TSize of 1 × 10^−8^ s (Table 6—Set 3). Figure 15 shows that the peak position shifted to an Erods parameter of 1.0 and a Soft factor of 1.0 and a slightly higher peak deceleration was achieved. Comparing Figure 14 and Figure 15 the TSize can be seen to be having an effect on the results independently of the Soft factor as there is a clear difference between the results even when the soft factor is set to 1.0 (no softening). High peaks are seen at the extremes of the Soft factors, full softening (0.0) and no softening (1.0).

Comparing the experimental and numerical results in Figure 13 it can be seen that the numerical models are over predicting the early deceleration and under predicting the later deceleration. It is thought that the increase in the TSize, which should cause more element deletion, has reduced the energy consumed in the initial deceleration of the nose cone model (reducing the deceleration) allowing a larger peak to occur later (Figure 15), which is more in line with the experimental results. To see if reducing the post-failure stresses could have a similar effect the Slim T parameter (that affects the tensile stress after tensile failure of either fiber or matrix) was investigated. The effect of the Erods parameter was also studied with the Soft factor set to zero (full softening) and Tsize set to 1 × 10^−8^ s (Table 6—Set 4).

Figure 16 shows less variation in deceleration between model runs than for Figure 15 but the peak deceleration is greater in Figure 15. To see what the effect of varying both the Soft factor and the Slim T parameters has a range of model were run with varying Soft, Erods and Slim T parameters, Figure 17, Figure 18, Figure 19, Figure 20 and Figure 21, with the Tsize set to 1 × 10^−11^ s (Table 6—Set 5). The selection of Erods parameters between 0.4 and 1.0 was based on earlier results that showed this region of Erods parameters produced the highest peak decelerations.

The highest peak deceleration was obtained with a slim T parameter of 0.2, Erods parameter of 0.4 and a Soft factor of 0.0. This deceleration was not as high as achieved with settings; all Slim factors equal 1.0, Soft equals 1.0, TSize equals 1 × 10^−8^ s and Erods equals 1.0, as seen in Figure 15. To determine whether decreasing the post-failure stresses would further increase the peak deceleration the Slim C and Slim S parameter (reduction in compressive stresses after failure in fiber or matrix and reduction in shear stress after shear stress failure) were reduced with the Slim T parameter set to 0.1. Figure 22 shows the effect on the rigidwall deceleration curve of changing the Slim C and Slim S parameters with various values of Erods and the soft factor set to 0.0 (Table 8—Set 6). This gave lower maximum peak decelerations than obtained previously (see Figure 15), and also caused the peaks to occur at a later rigidwall displacement than that found experimentally.

To see whether a combination of different post-failure stresses could give the desired reduction in initial over prediction of deceleration the tensile (SlimT), compressive (SlimC) and shear (Slim S) slim factors were all adjusted. The TSize parameter, Erods parameter and Soft factor were all set to zero (Table 8—Set 7). The Soft factor has no effect as the TSize parameter was set to zero. These settings do not allow for element deletion or softening so the effect of post-failure reduction of stresses was investigated independently.

The results for the various combinations of Slim factors was not as good as previous results when only the Slim T factor was reduced (see Figure 23). The best result was still found to be for the case where there was no post-failure reduction in material stresses (All Slim equal 1.0, Soft equals 1.0, TSize equals 1 × 10^−8^ and Erods equals 1.0, as seen in Figure 15). To see whether a better simulation could be obtained in the region of the best fit, so far, a narrower range of Soft factors were used (0.6 to 1.0) with TSize set to 1 × 10^−7^ s, 1 × 10^−8^ s and 1 × 10^−9^ s. The Erods parameters were varied from 0.0 to 1.0, the all Slim factors set between 0.6 to 1.0 and the soft factor set to 1.0 (no softening of elements in front of the crash front) (Table 8—Set 8). The results were not as high as the highest peak achieved previously (Figure 15). In Figure 24 the results for a TSize of 1 × 10^−9^ s have been presented, as this TSize gave the highest peak deceleration.

The material properties were derived from data from other papers and so there is some level of uncertainty in terms of the actual material properties. To see if variations might have a significant effect the failure strengths and moduli of the material were reduced by 10%. In each case either all the moduli were reduced by 10% or all the failure strengths were reduced by 10% or both the moduli and strengths were reduced by 10%. To ensure just the effect of reduced material properties was investigated all the Slim factors were set to 1.0, Erods was set to 0.0 and TSize was set to 0.0 (Table 8—Set 10). The results are shown in Figure 25.

Figure 25 shows that reducing the material strength and stiffness does not have a significant effect on the magnitude and position of the peak deceleration. It is known that the amount of integration points across the element and the addition of hourglass viscosity can affect the bending stiffness of the elements. As the loading on the nose cone is initially perpendicular to the cone surface the initial results are going to be more sensitive to the bending stiffness of the elements. As integration points and hourglassing viscosity are known to influence the bending stiffness of shell elements some models were run to check their effect. 3 to 5 integration points are recommended for non-linear materials [30]. To increase the amount of integration points across the element the laminates were subdivided to ensure that there were 12 integration points across the thickness of every shell element. Hourglassing viscosity was also added using a coefficient value of 0.03 and local shell coordinates were employed (invariant node numbering, INN = 2). The results of adding invariant node numbering and integration points did not significantly change the results but adding the hourglassing viscosity did reduce the initial over prediction but also reduced the peak prediction (similar effect to that of modulus reduction seen in Figure 25). To examine the effect of mesh sensitivity two models with higher density meshes were run. The model parameters were set the same as the model that achieved the highest peak deceleration in Figure 15 (All Slim factors set to 1.0, TSize set to 1 × 10^−8^ s, Erods set to 1.0 and the Soft factor set to 1.0). The results for the three mesh sizes can be seen in Figure 26. 

There appeared to be some run-to-run variation within the models. To investigate this the input file that had produced the best result in Figure 15 (Slim factors set to 1.0, TSize set to 1 × 10^−8^ s, Erods set to 1.0 and Soft factor set to 1.0) was run thirteen times. The results from these model runs plus the original model run are shown in Figure 27.

There is considerable run to run variation seen in Figure 27 which makes it much more difficult to identify the best parameter fit. To check that certain parameter settings have not been discarded prematurely a series of repeat runs (five) were carried out where the Failure Surface, Soft, Erods and Tsize values were varied. The Soft factor was set to either 0.0 or 1.0, as these extremes generated the best results previously. The Erods parameter was set to either 0.0 or 1.0. An Erods parameter of 1.0 was chosen as this previously generated good results and the value of 0.0 was chosen to see whether having an Erods parameter affected the run-to-run variability within the models. The failure surface (FS) was investigated again just to ensure that the run-to-run variation had not caused the smooth failure surface to be rejected prematurely. The Slim factors were all set to 1.0. Table 10 shows the mean value for each set of model runs with the standard deviation.

There is considerable run-to-run variation within the models in Table 10 but the results are in line with previous results giving confidence in the process used for discerning the best fit parameters. There were though some high peaks achieved when a Slim T of 0.2 and 1.0 was used which may have been affected by the run-to-run variation. To check if run to run variation might make these results higher the best previous models were run five times. The means and standard deviations for the results from these model runs is show in Table 11.

Although the best individual result was achieved using TSize = 1 × 10^−8^ s and Soft = 1.0 (Figure 15) the best average peak deceleration result was found using the same parameter settings but with TSize = 1 × 10^−9^ s and Soft = 0.0 (Table 10, Set 12).

The deformation results for the model run that achieved the highest peak deceleration (Slim factors set to 1.0, TSize set to 1 × 10^−9^ s, Erods set to 1.0 and Soft factor set to 0.0) is shown in Figure 28, Figure 29 and Figure 30. Figure 28 shows the deformation of the nose cone at different times during the impact test. It can be seen that after 0.05 s the rigidwall starts to rebound. Figure 29 shows the permanent deformation after the impact at 0.1 s. Figure 30 shows the elements that have exceeded the failure criteria in the longitudinal (Tensile/Compressive), transverse (Tensile/Compressive) and shear directions.

## 5. Discussion

**Figure 12:**
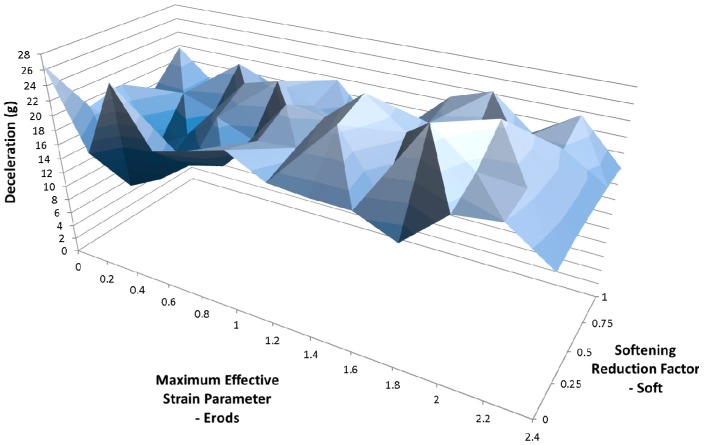
Effect of Erods parameter and Soft factor with All Slim = 1.0, TSize = 1 × 10^−11^ and FS = 1.0.

In Figure 11 it can be seen that the non-faceted surface model (FS = 1) overestimates the amount of deceleration during the initial 0.23 m of displacement and then substantially underestimates the experimental peak deceleration (56 g at 0.253 m). The maximum deceleration of 27.3 g occurs when Soft = 0.25 and Erods = 1.8 (see Figure 12). This value is well below the experimental peak and also occurs at a further rigidwall displacement position than the experimental peak. The underestimation of peak deceleration when using a smooth surface (FS = 1) was further confirmed when examining run-to-run model variations, as shown in Table 10.

**Figure 13:**
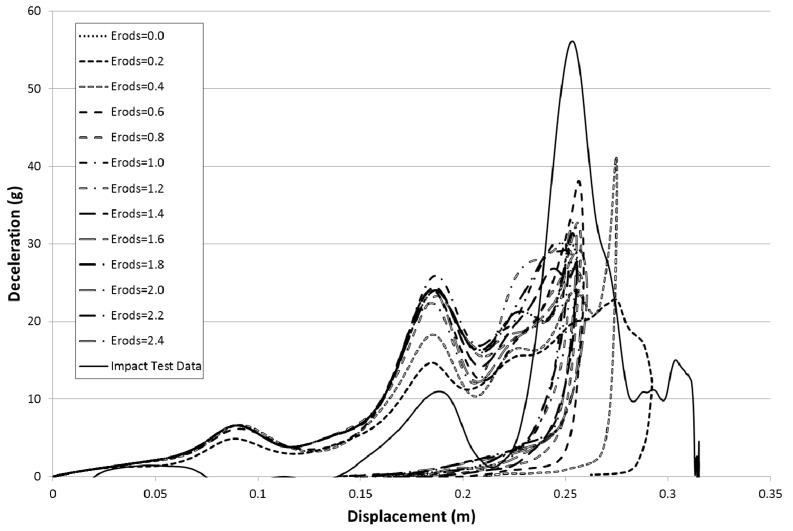
Effect of Erods parameter with All Slim = 1.0, TSize = 1 × 10^−11^, Soft = 1.0 and FS = −1.0.

**Figure 14:**
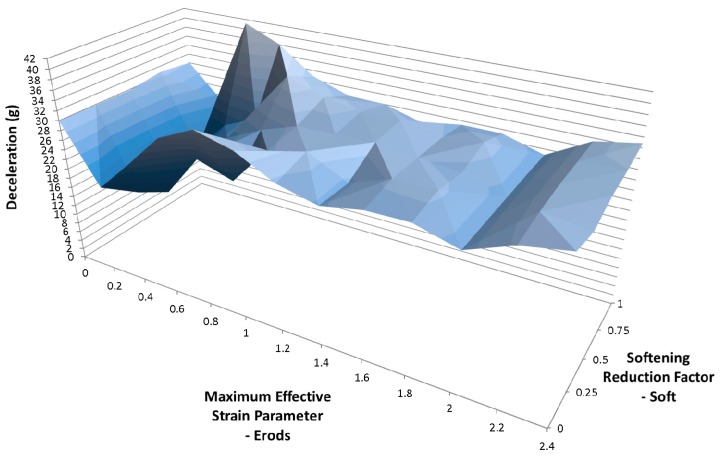
Effect of Erods parameter and Soft factor with All Slim = 1.0, Size = 1 × 10^−11^ and FS = −1.0.

When the failure surface is changed to a faceted surface (FS = −1) much higher decelerations are obtained (see Figure 13), although again the model overestimates the early deceleration and underestimates the later deceleration. The maximum deceleration of 41.2 g shown in Figure 14 occurs when Soft = 1.0 and Erods = 0.4, at a displacement of 0.275 m. This is closer in terms of magnitude to the experimental data but occurs at a later displacement then the experimental peak. Both failure surfaces are stated to be suited for woven fabrics but the faceted failure surface gave significantly higher peak decelerations.

**Figure 15:**
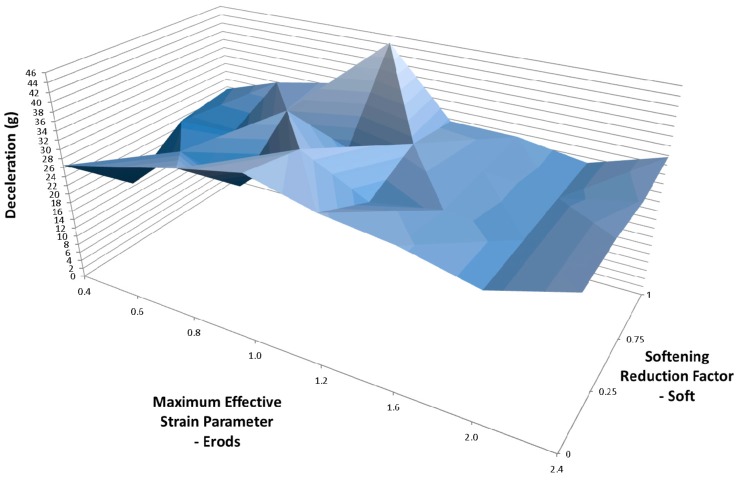
Effect of Erods parameter and Soft factor with All Slim = 1.0, TSize = 1 × 10^−8^ and FS = −1.0.

Changing the TSize to 1 × 10^−8^ s shifted the position of the maximum peak deceleration on the plot surface to an Erods parameter of 1.0 (Figure 15). The TSize parameter affects the time-step size at which elements are deleted. The idea is that the TSize should be set low enough that it will not detrimentally affect the component strength while preventing long run times that can occur due to element distortion. The deceleration value using Erods = 1.0 and Soft = 1.0 was found to be 44.4 g, occurring at a displacement of 0.243 m. This improvement, due to TSize change, seemed counterintuitive as it would be expected to weaken the component (earlier element deletion) and reduce the decelerations along the majority of the length of the cone. This would therefore be expected to reduce the peak deceleration within the 0.23 m to 0.29 m displacement region of interest, where it occurs experimentally. This would then result in very high late peak deceleration due to crushed material getting compressed between the nose cone supports and the rigid wall. In the models the deceleration did reduce as expected in the early stage of the impact, leaving more kinetic energy to be absorbed later in the impact, but this did not result in a late high peak but actually increased the peak deceleration within the region of interest.

**Figure 16:**
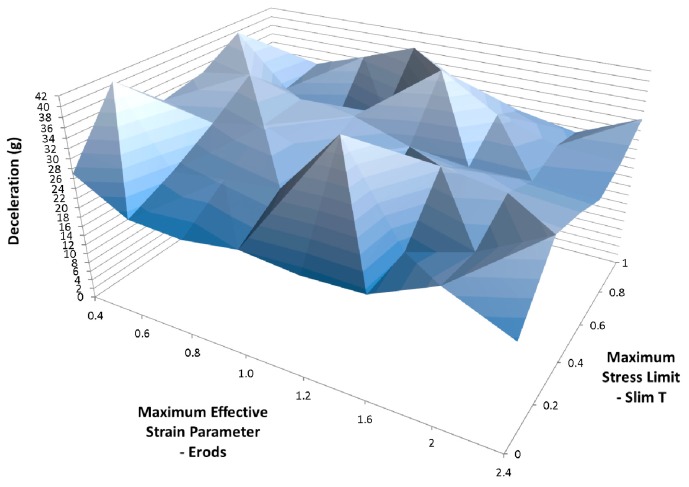
Effect of Erods parameter and Slim T parameter with Soft = 0.0, Slim C = 1.0, Slim S = 1.0, TSize = 1 × 10^−8^ and FS = −1.0.

**Figure 17:**
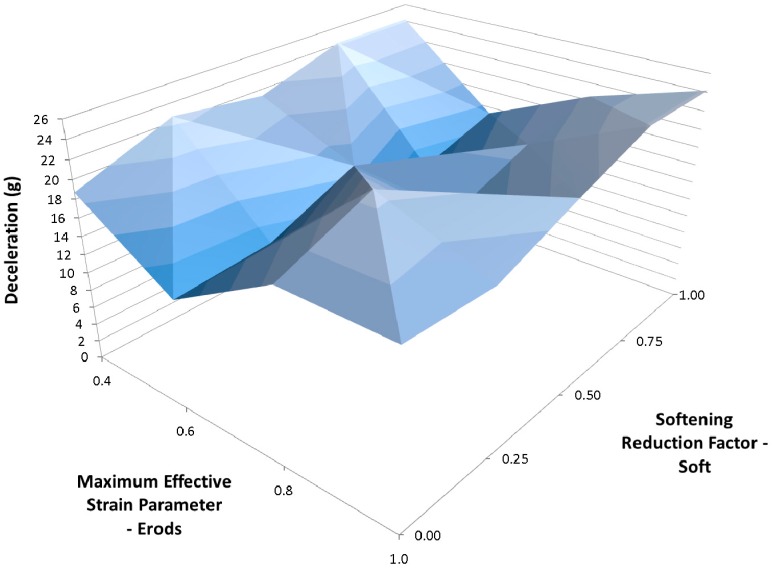
Effect of Erods parameter and Soft factor with Slim T = 0.0, Slim C = 1.0, Slim S = 1.0, TSize = 1 × 10^−11^ and FS = −1.0.

To see whether reducing the post-failure stress in elements would have a similar effect, various Slim T parameter values (fiber and matrix tensile post-failure stress) were examined. The effect of Erods was also investigated with the Soft factor set to zero (full softening). The maximum deceleration obtained was found at an Erods parameter of 1.2 and Slim T parameter of 0.2, as shown in Figure 16. The deceleration at this point on the surface plot is 40.6 g occurring at a rigidwall displacement of 0.26 m. Although the maximum deceleration value is not as high as in previous runs the surface values overall are higher than previous (Figure 15).

**Figure 18:**
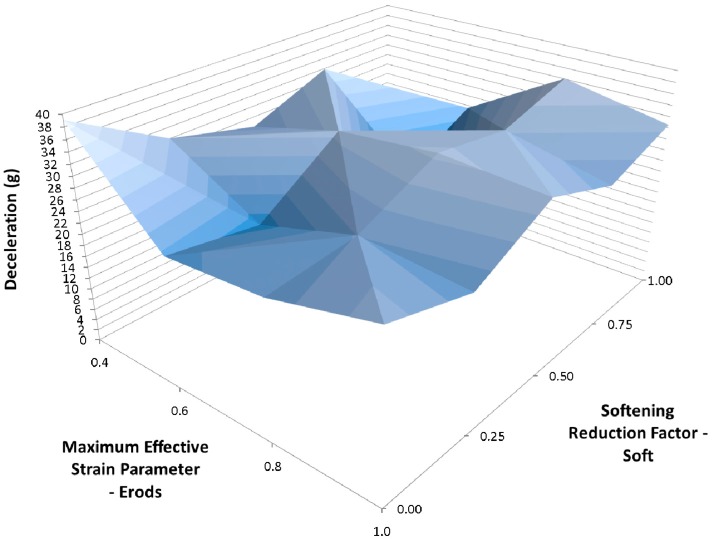
Effect of Erods parameter and Soft factor with Slim T = 0.2, Slim C = 1.0, Slim S = 1.0, TSize = 1 × 10^−11^ and FS = −1.0.

**Figure 19:**
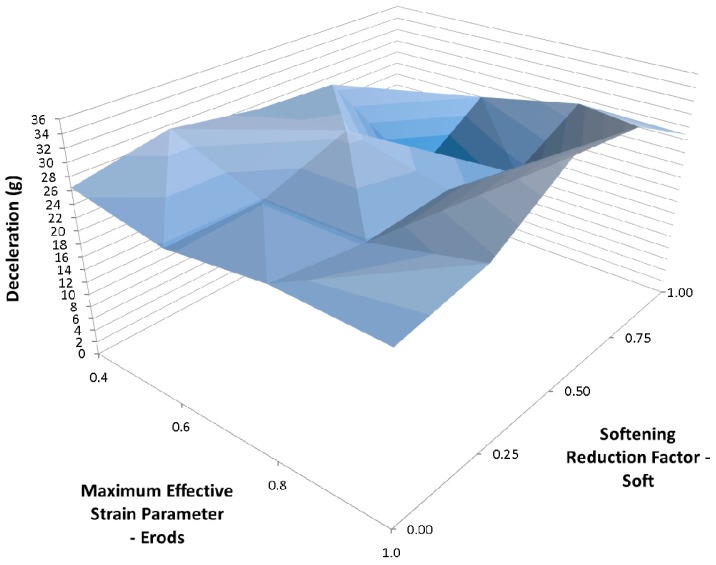
Effect of Erods parameter and Soft factor with Slim T = 0.4, Slim C = 1.0, Slim S = 1.0, TSize = 1 × 10^−11^ and FS = −1.0.

**Figure 20:**
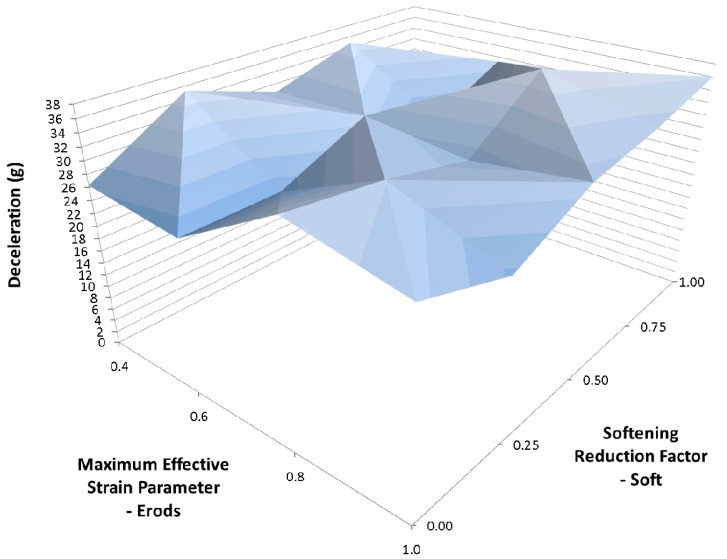
Effect of Erods parameter and Soft factor with Slim T = 0.6, Slim C = 1.0, Slim S = 1.0, TSize = 1 × 10^−11^ and FS = −1.0.

**Figure 21:**
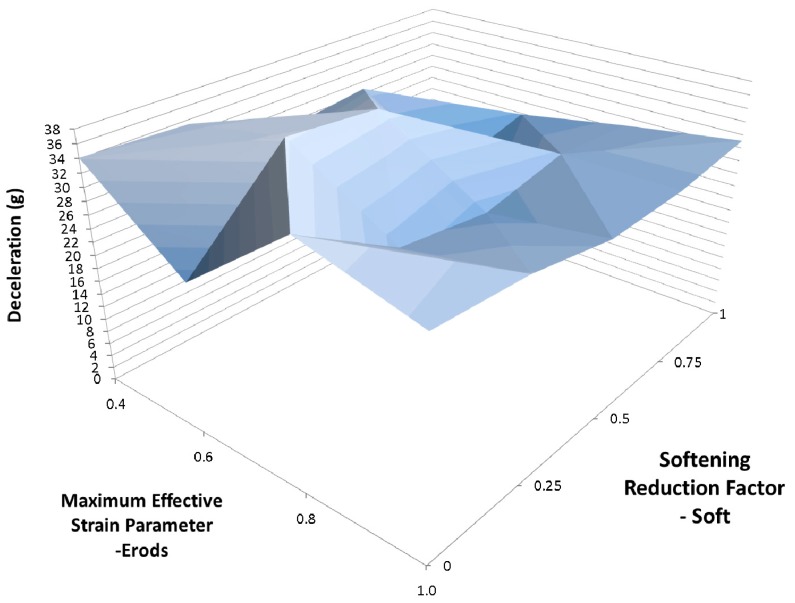
Effect of Erods parameter and Soft factor with Slim T = 0.8, Slim C = 1.0, Slim S = 1.0, TSize = 1 × 10^−11^ and FS = −1.0.

**Figure 22:**
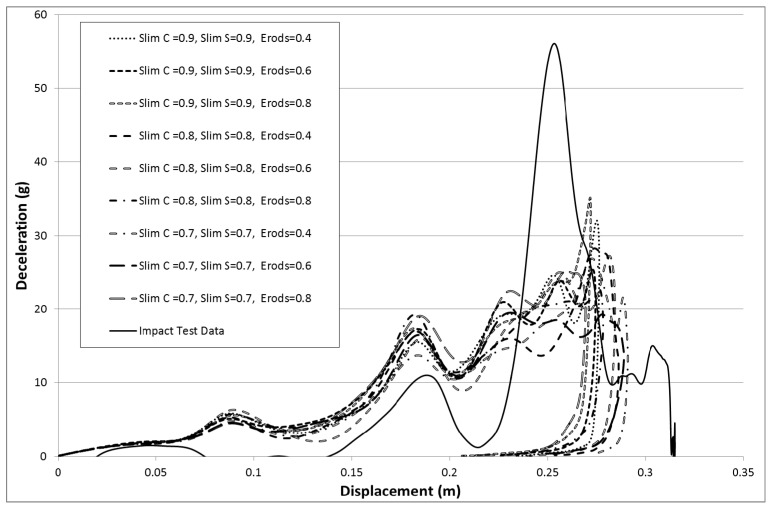
Effect of Slim C, Slim S and Erods parameters with Slim T = 0.1, TSize = 1 × 10^−11^, Soft = 0.0 and FS = −1.0.

To investigate whether the Soft factor influences the impact of the lower Slim T parameter values, several models runs were carried out where both the Soft factor and the Slim T parameters were varied (see Figure 18, Figure 19, Figure 20, Figure 21 and Figure 22). The TSize was set to 1 × 10^−11^ s to reduce the effect of element deletion on the results. No strong influence was found in terms of the Soft factor. The highest deceleration of 39 g was obtained for a Slim T parameter of 0.2, an Erods parameter of 0.4 and a Soft factor of 0.0 (see Figure 18). This peak deceleration was at a displacement position of 0.275 m.

To see whether reducing the post-failure compressive and shear stresses as well as the post-failure tensile stress could generate the initial drop in impact deceleration (to help generate a later higher peak), the Slim C and Slim S values were varied while keeping the Slim T parameter at 0.1. Although this did reduce the early overestimation of deceleration (see Figure 22 vs. Figure 13) it also reduced the deceleration at the peak position as well. The energy during the later stages of the impact can be seen to be dissipated over a longer displacement (seen by the model peak deceleration shifting further along than the experimental peak deceleration) which will be due to an over reduction of the structural stiffness of the cone. This is problematic as this indicates that the models have nose cone stiffnesses at the start of the impact that are too hard but at the end of the impact are too soft.

**Figure 23:**
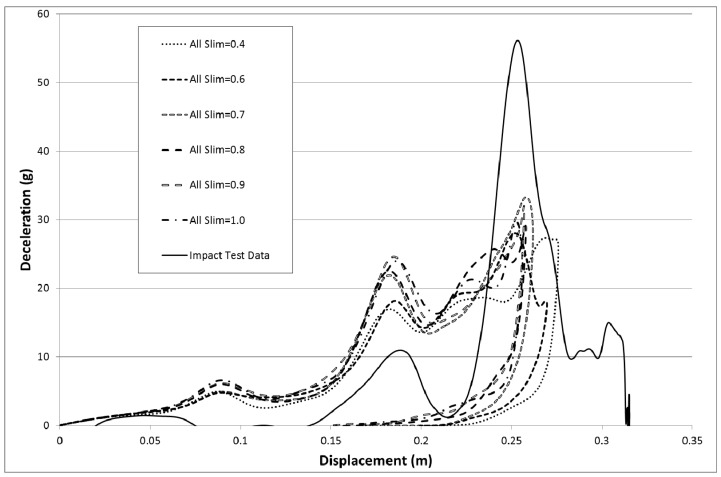
Effect of Slim factors with TSize = 0.0, Erods = 0.0, Soft = 0.0 and FS = −1.0.

To determine if the low value of Slim T was detrimental it was decided to investigate changing all the Slim factors by the same amount. Figure 23 show that this had the negative effect of increasing the early deceleration but had the benefit of increasing the peak deceleration, but not up to the level of earlier runs.

**Figure 24:**
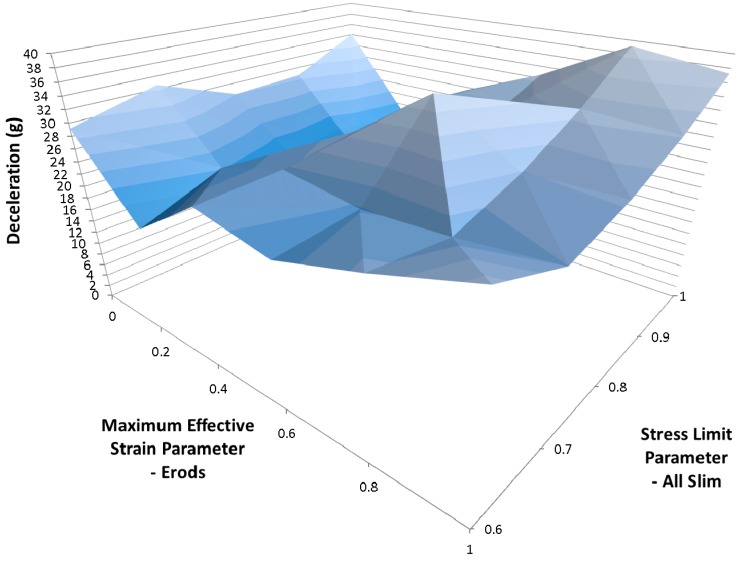
Effect of Erods and Slim factors with T Size = 1 × 10^−9^, Soft = 1.0 and FS = −1.0.

To investigate the effect of TSize as well as post-failure softening it was decided to use three different TSize values with a Soft value of 1.0 (no softening of crash front) and the Erods and Slim factors set between 0.0 to 1.0 and 0.6 to 1, respectively. The generated surface plots were similar to each other but lower TSize’s gave slightly better results. The highest deceleration of 39.2 g was achieved with a TSize of 1 × 10^−9^ and an Erods parameter of 0.8 (see Figure 24).

**Figure 25:**
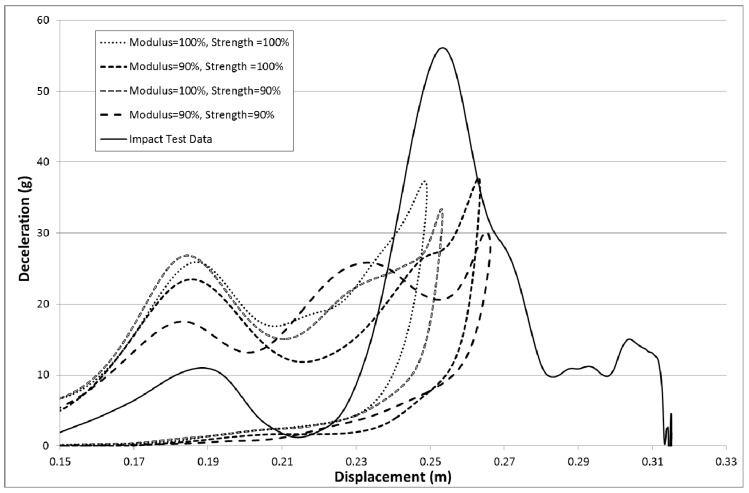
Effect of reducing modulus and failure strengths by 10% with All Slim = 1.0, TSize = 0.0, Erods = 0.0, Soft = 0.0 (no softening as TSize = 0.0) and FS = −1.0.

As the material properties were gathered from papers, and seeing that post-failure strength had an effect, it was decided to investigate whether small changes in strength or moduli properties might improve the results. Figure 25 shows curves for a 10% reduction in the moduli values, a 10% reduction in strength values and 10% reduction in moduli and strength values. The 10% reduction in strength increased the peak deceleration, but not significantly. Reducing the moduli did create a more substantial increase in peak deceleration but shifted the displacement of the peak away from the experimental peak position. Reducing both moduli and strength caused a peak displacement shift and resulted in a negligible increase in peak deceleration. It did though reduce the initial over-prediction of deceleration during the early stages of the impact.

It should be noted that the initial loading of the nose cone is perpendicular to the nose cone surface and as the impact progresses the loading becomes more parallel to the nose cone surface. The cone would be expected to fold relatively easily during the initial perpendicular loading and therefore be quite weak. Experimentally and numerically the cone is weak initially but there is significant numerical overestimation of the impact deceleration. This indicates that the material is not folding as easily numerically as it does experimentally, which may be due to the model over predicting the bending stiffness of the cone. Element type, number of integration points across the element thickness, hour glassing viscosity model, hour glassing coefficient and material properties are known to affect the bending stiffness predicted by the model. Changing the strength and modulus of the material properties, the amount of integration points and adding hourglassing viscosity did not though have a significant effect on reducing the early overestimation of the deceleration. Investigating the effect of element type and element parameters is suggested as an area for further work.

**Figure 26:**
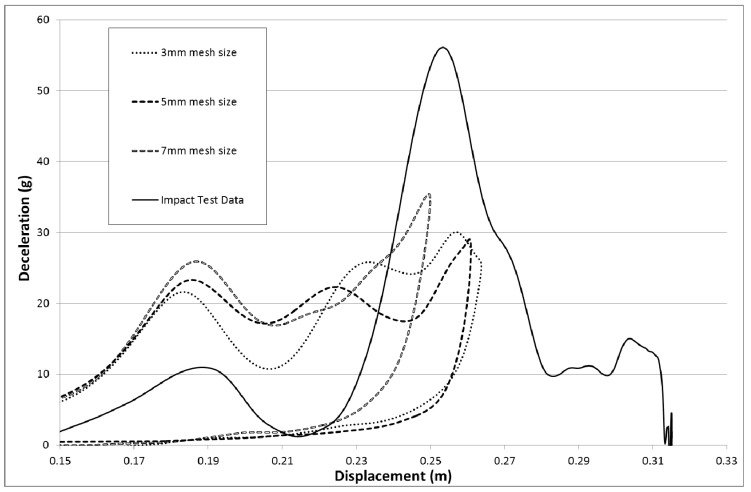
Investigation of mesh sensitivity with three runs using the same model input file with All Slim = 1.0, TSize = 1 × 10^−8^, Erods = 1.0, Soft = 1.0 and FS = −1.0.

To check the effect of mesh sensitivity three models with identical parameters were run using 3 mm, 5 mm and 7 mm element sizes (see Figure 26). Although higher mesh density models gave poorer predictions of experimental rigidwall deceleration a full parametric study, similar to that carried out for the 7 mm element mesh, would need to be carried out to fully see the effect of increased mesh density.

**Figure 27:**
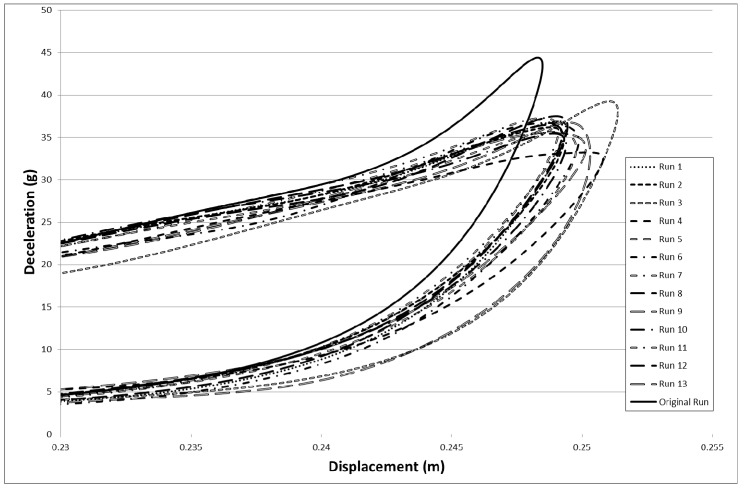
Investigation of time-step sensitivity with fourteen runs using the same model input file with All Slim = 1.0, TSize = 1 × 10^−8^, Erods = 1.0, Soft = 1.0 and FS = −1.0.

**Figure 28:**
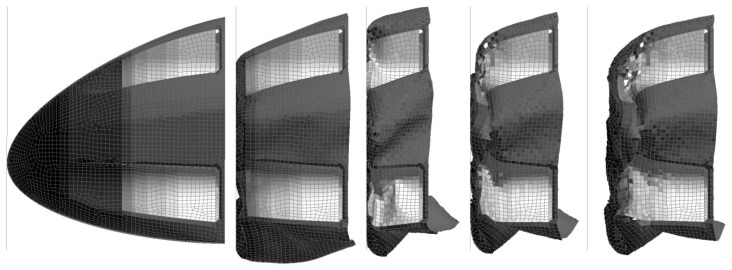
D3Plot images of wall hitting nose cone at 0, 0.25, 0.5, 0.75 and 0.1 s of LS-Dyna model with settings All Slim = 1.0, TSize = 1 × 10^−9^, Erods = 1.0, Soft = 0.0 and FS = −1.0.

**Figure 29:**
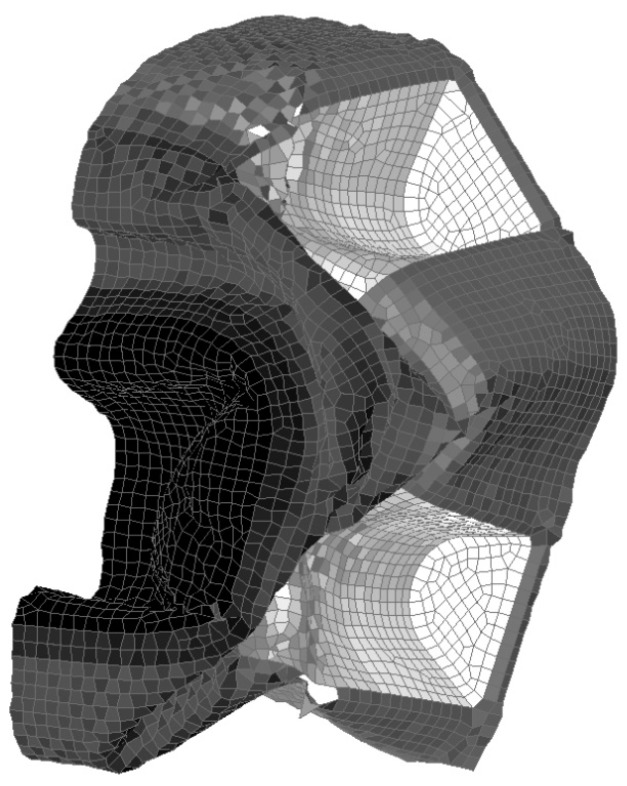
D3Plot image of damage after impact of LS-Dyna model with settings All Slim = 1.0, TSize = 1 × 10^−9^, Erods = 1.0, Soft = 0.0 and FS = −1.0.

When running some repeat models, it became clear that runs using the same input file could generate slightly different deceleration displacement curves. To check this, repeated runs of the model that had given the highest initial peak deceleration were carried out, as shown in Figure 27. As can be seen there is some divergence in the curves. This is probably due to time-step size variations, which are generated by slight hardware differences on the computer nodes used on the high-performance computing facility. There is no discussion of this with respect to explicit numerical codes but there is work using implicit codes showing that carbon composite damage progressive models are time-step sensitive [31].

To check that the run-to-run variation had not given poor parameter fit values, a series of repeated runs of identical models were made, as shown in Table 10. The Soft factor and Erods parameter were set to either 0.0 or 1.0. These Soft factors plus the Erods parameter of 1.0 produced good previous results. Setting the Erods parameter to 0.0 was done to check whether reducing the amount of element deletion would produce less run-to-run variation. The two Failure surfaces were also compared. There was lower variability in the smooth surface failure results (FS = 0) but this was due to getting low decelerations within the set displacement range, caused by the main model peak falling outside the set displacement range (nose cone too soft in the model). Setting the Erods parameter to 0.0 did reduce the variability in results, for the faceted surface, but gave lower results. These runs gave similar results to earlier, giving confidence in the parametric approach. To check that three other high result models were not discarded prematurely they were also repeated five times (see Table 11), but were found to give lower average results than their previous results. The best results were obtained when the Soft factor was set either to 0.0 or 1.0, the Slim factors were set to 1.0, the Tsize set to around 1 × 10^−8^ or 1 × 10^−9^ s and the Erods parameter set close to 1.0.

The run times for most models took between 30 to 120 min using 8 cores and 64 GB of memory on each cpu node on a Linux based Dell Poweredge server. Models with low TSize values (1 × 10^−11^ or 1 × 10^−10^ s) could take up to 24 h to run and would sometimes fail to complete in the maximum time set (48 h).

**Figure 30:**
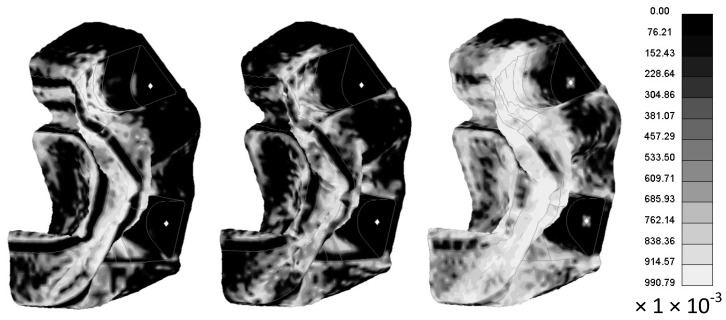
D3Plot images of longitudinal, transverse and shear damage (respectively) after impact of LS-Dyna model with settings All Slim = 1.0, TSize = 1 × 10^−9^, Erods = 1.0, Soft = 0.0 and FS = −1.0 (Black undamaged: 0, White fully damaged: 1).

The extent of damage, seen in Figure 30, for the model that best fitted the rigidwall deceleration is similar to that found experimentally, with the maximum failure in the regions that fragmented during the experiment. Progressive failure criteria, as used in this work, soften the material rather than fragment the material. It is possible through the Erods and Tsize criteria to delete elements but this is typically used to prevent the model becoming unstable, due to excessive element deformation, rather than trying to replicate the extent of visual damage. In this case, for the model with the best rigidwall deceleration fit, there is limited erosion of elements, as seen in Figure 30. This is a limitation of using progressive damage failure criteria.

## 6. Conclusions

The general shape of the deceleration displacement curves match reasonably well when the faceted failure surface option (FS = −1) is selected within the options for material 58 within LS Dyna. The initial deceleration tends to be overestimated while the peak deceleration tends to be underestimated.

Varying parameters such as Soft, Erods, TSize and Slim has some effect on increasing the peak deceleration but there was no solution that gave high peaks at the experimental high peak displacement position. Generally setting the Soft factor to either 0.0 or 1.0, the Slim factors to 1.0, the Tsize around 1 × 10^−8^ or 1 × 10^−9^ s and the Erods parameter around 1.0 was shown to give the best experimental fit. The models displayed mesh sensitivity and run-to-run variation for identical models, which indicates time-step sensitivity as well. The highest peak deceleration obtained numerically, within the rigidwall displacement range of 0.23 to 0.29 m, was about 30% lower than the experimental value.

The run times of the models were reasonably short but the large number of runs meant that the sum total time to run the models was considerable.

## Figures and Tables

**Table 1:** Mesh Parameters.

Mesh Name	Element Size	Automatic Mesh Capture Size	Element Type
Mesh_7 mm	7 mm	3.6 mm	Linear Quad
Mesh_5 mm	5 mm	2.6 mm	Linear Quad
Mesh_3 mm	3 mm	1.6 mm	Linear Quad

**Table 2:** Carbon-epoxy prepreg woven properties.

Property	CF3202/MTM57 [23]	CFS003/LTM25 [25]	Material Properties Used in the Model
Cloth specific weight	245 g/m^2^	-	-
Volume fraction	-	46.9%	-
Density	-	1453 kg/m^3^	1408 kg/m^3^
Young’s tensile modulus in longitudinal direction *E*_11_*^t^*	64.2 × 10^9^ Pa	48.7 × 10^9^ Pa	64.6 × 10^9^ Pa
Young’s tensile modulus in longitudinal direction *E*_11_*^c^*	-	49.64 × 10^9^ Pa	-
Young’s tensile modulus in transverse direction *E*_22_*^t^*	65.1 × 10^9^ Pa	51.8 × 10^9^ Pa	64.6 × 10^9^ Pa
Young’s compressive modulus in transverse direction *E*_22_*^c^*	-	54.1 × 10^9^ Pa	-
Poisson’s ratio *ν*_12_	0.05	0.042	-
Poisson’s ratio *ν*_21_	0.05	0.035	0.05
Shear modulus *G*_12_	-	2.85 × 10^9^ Pa	2.85 × 10^9^ Pa
Shear modulus *G*_23_	-	-	2.85 × 10^9^ Pa
Shear modulus *G*_31_	-	-	2.85 × 10^9^ Pa
Longitudinal ultimate tensile stress *σ*_11_*^ut^*	642 × 10^6^ Pa	562.6 × 10^6^ Pa	642 × 10^6^ Pa
Longitudinal ultimate compressive stress *σ*_11_*^uc^*	-	641.9 × 10^6^ Pa	642 × 10^6^ Pa
Transverse ultimate tensile stress *σ*_22_*^ut^*	665 × 10^6^ Pa	612.3 × 10^6^ Pa	665 × 10^6^ Pa
Transverse ultimate compressive stress *σ*_22_*^uc^*	-	563.3 × 10^6^ Pa	563 × 10^6^ Pa
In plane ultimate shear stress *τ*_12_*^uIP^*	-	84.12 × 10^6^ Pa	84 × 10^6^ Pa
Interlaminar ultimate shear stress *τ*_12_*^uIL^*	71.5 × 10^6^ Pa	-	-

**Table 3:** Additional material parameters set within the model [19,29].

Material Parameter Set	Meaning
Slim T (Slim1T & Slim2T)	Factor to determine the minimum tensile stress limit after tensile failure (fiber and matrix can be set separately but were kept the same). A value of one will prevent stress reduction and a value of zero will reduce the stress to zero.
Slim C (Slim1C & Slim2C)	Factor to determine the minimum compressive stress limit after compressive failure (fiber and matrix can be set separately but were kept the same). A value of one will prevent stress reduction and a value of zero will reduce the stress to zero.
Slim S (SlimS)	Factor to determine the minimum shear stress limit after shear stress failure. A value of one will prevent stress reduction and a value of zero will reduce the stress to zero.
TSize	Time step for automatic element deletion. The crashfront-algorithm is started if a value for TSIZE is input (Soft Factor).
Erods	Maximum effective strain for element layer deletion. A value of unity equals a 100% strain.
Soft	Reduces material strength in elements immediately behind the crash front. The strength reduction increases as the value goes from one (no reduction) to zero [ 17].
FS	EQ.1.0: smooth failure surface; EQ.−1.0: faceted failure surface.

**Table 4:** Parameters for control section of model.

Control Card	Control Item	Value Set	Effect
Shell	ESORT	1	Assigns triangular element formulation (Elform 4) to unassigned triangular elements.
	LAMSHT	1	Corrects for assumption of uniform transverse strain through shell thickness.
Termination	ENDTIM	0.1	Sets the maximum run time for the model.

**Table 5:** Additional control section parameters.

Hourglass	IHQ	4	Viscosity added to shell elements to prevent hourglassing. A value of 4 utilizes the stiffness form of Flanagan-Belytschko.
	QH	0.03	Hourglass coefficient.
Accuracy	INN	2	When set to 2 invariant node numbering is applied to shell and thick shell elements.

**Table 6:** Contact friction values.

Friction Variables	Value Set	Effect
FS	0.25	Sets the static friction value
FD	0.2	Sets the dynamic friction value

**Table 7:** Parameter used in different model runs (Part 1).

Parameter	Set 1	Set 2	Set 3	Set 4	Set 5
Element Size	7 mm	7 mm	7 mm	7 mm	7 mm
Soft	0.0–1.0 (0.25 steps)	0.0–1.0 (0.25 steps)	0.0–1.0 (0, 0.25, 075, 1.0)	0.0	0.0–1.0 (0.25 steps)
Slim T	1.0	1.0	1.0	0.0–1.0 (0.2 Steps)	0.0–0.8 (0.2 Steps)
Slim C	1.0	1.0	1.0	1.0	1.0
Slim S	1.0	1.0	1.0	1.0	1.0
Erods	0–2.4 (0.2 Steps)	0–2.4 (0.2 Steps)	0–2.4 (0.2 steps)	0.4–1.0 (0.2 steps), 1.2–2.4 (0.4 steps)	0.4–1.0 (0.2 steps)
TSize	1 × 10^−11^	1 × 10^−11^	1 × 10^−8^	0.0	1× 10^−11^
Modulus	Normal	Normal	Normal	Normal	Normal
Strength	Normal	Normal	Normal	Normal	Normal
FS	−1.0	1.0	−1.0	−1.0	−1.0

**Table 8:** Parameter used in different model runs (Part 2).

Parameter	Set 6	Set 7	Set 8	Set 9	Set 10
Element Size	7 mm	7 mm	7 mm	3, 5 and 7 mm	7 mm
Soft	0.0	0.0	1.0	0.0	1.0
Slim T	0.1	0.4, 0.6. 0.7. 0.8, 0.9, 1.0	0.6–1 (0.1 Steps)	1.0	1.0
Slim C	0.7–0.9 (0.1 steps)			1.0	1.0
Slim S	1.0			1.0	1.0
Erods	0.4–0.8 (0.2 steps)	0.0	0–1 (0.2 Steps)	0.0	1.0
TSize	1 × 10^−11^	0.0	1 × 10^−7^, 1 × 10^−8^, 1 × 10^−9^	0.0	1× 10^−8^
Modulus	Normal	Normal	Normal	Normal	90–100% of Normal
Strength	Normal	Normal	Normal	Normal	90–100% of Normal
FS	−1.0	−1.0	−1.0	−1.0	−1.0

**Table 9:** Parameter used in different model runs (Part 3).

Parameter	Set 11	Set 12	Set 13	Set 14
Element Size	7 mm	7 mm	7 mm	7 mm
Soft	0.0	0.0	1.0	1.0
Slim T	1.0	1.0	1.0	1.0
Slim C	1.0	1.0	1.0	1.0
Slim S	1.0	1.0	1.0	1.0
Erods	0.0	1.0	0.0	1.0
TSize	1 × 10^−7^, 1 × 10^−8^, 1 × 10^−9^, 1 × 10^−10^, 1 × 10^−11^	1 × 10^−7^, 1 × 10^−8^, 1 × 10^−9^, 1 ×10^−10^, 1 × 10^−11^	1 × 10^−7^, 1 × 10^−8^, 1 × 10^−9^, 1 ×10^−10^, 1 × 10^−11^	1 × 10^−7^, 1 × 10^−8^, 1 × 10^−9^, 1 ×10^−10^, 1 × 10^−11^
Modulus	Normal	Normal	Normal	Normal
Strength	Normal	Normal	Normal	Normal
FS	1.0, −1.0	1.0, −1.0	1.0, −1.0	1.0, −1.0

**Table 10:** Mean and standard deviations (St Dev) for peak deceleration values (0.23 m < wall displacement < 0.29 m), for parameter sets 11 to 14, based on five runs for each given value.

**Smooth Failure Surface (FS = 1)**								
**Tsize**	**Set 11**		**Set 12**		**Set 13**		**Set 14**	
	**Soft = 0.0, All Slim = 1.0, Erods = 0.0**		**Soft = 0.0, All Slim = 1.0, Erods = 1.00**		**Soft = 1.0, All Slim = 1.0, Erods = 0.0**		**Soft = 1.0, All Slim = 1.0, Erods = 1.0**	
	**Mean**	**St Dev**	**Mean**	**St Dev**	**Mean**	**St Dev**	**Mean**	**St Dev**
1 × 10^−7^	8.53	1.59	21.67	0.03	20.22	1.35	21.67	0.03
1 × 10^−8^	20.20	1.28	21.66	0.05	21.32	2.25	21.28	0.90
1 × 10^−9^	19.94	1.69	21.50	0.37	17.36 *	2.81 *	21.31	2.04
1 × 10^−10^	19.84	1.35	21.68 *	0.02 *	20.03	1.30	22.00	0.73
1 × 10^−11^	19.75	1.19	21.67	0.04	19.34	0.69	21.67	0.03
**Facetted Failure Surface (FS = −1)**								
**Tsize**	**Set 11**		**Set 12**		**Set 13**		**Set 14**	
	**Soft = 0.0, All Slim = 1.0, Erods = 0.0**		**Soft = 0.0, All Slim = 1.0, Erods = 1.0**		**Soft = 1.0, All Slim = 1.0, Erods = 0.0**		**Soft = 1.0, All Slim = 1.0, Erods = 1.0**	
	**Mean**	**St Dev**	**Mean**	**St Dev**	**Mean**	**St Dev**	**Mean**	**St Dev**
1 × 10^−7^	30.36	0.65	34.95	3.97	29.82	0.17	36.20	2.53
1 × 10^−8^	29.62	0.19	36.84	3.33	30.03	0.70	38.55	2.20
1 × 10^−9^	30.17	0.59	39.99	2.66	30.29	1.08	35.84	3.15
1 × 10^−10^	31.50	1.99	36.05	2.47	30.70	2.02	35.13	3.58
1 × 10^−11^	29.95	0.42	34.85	2.74	30.38	0.67	34.02	1.84

* Only four runs were used for these values (due to non-completion of model runs).

**Table 11:** Mean and standard deviations (St Dev) for peak deceleration values (0.23 m < wall displacement < 0.29 m) based on five runs of previous high peak models.

Facetted Failure Surface (FS = −1), Slim C = 1.0, Slim S = 1.0						
Tsize	Soft = 1.0, Erods = 0.6, Slim T = 1.0 (Figure 13)		Soft = 0.0, Erods = 1.2, Slim T = 0.2 (Figure 16)		Soft = 0.0, Erods = 0.4, Slim T = 0.2 (Figure 18)	
	Mean	St Dev	Mean	St Dev	Mean	St Dev
1 × 10^−11^	28.06	2.33	29.95	3.96	30.00	3.18

## Abbreviations

11, 22, 33	Indices representing normal material property directions.
12, 13, 23, 21, 31 and 32	Indices representing shear material property directions
*τ*_12_*^uIP^*	In plane ultimate shear stress
*τ*_12_*^uIL^*	Interlaminar ultimate shear stress
*σ*_11_*^ut^*	Longitudinal ultimate tensile stress
*σ*_11_*^uc^*	Longitudinal ultimate compressive stress
*σ*_22_*^ut^*	Transverse ultimate tensile stress
*σ*_22_*^uc^*	Transverse ultimate compressive stress
*ν*	Poisson’s ratio
CDM	Continuum Damage Mechanics
CFRP	Carbon Fiber Reinforced Plastic
*E*	Young’s modulus
*E*_11_*^t^*	Young’s tensile modulus in longitudinal direction
*E*_11_*^c^*	Young’s tensile modulus in longitudinal direction
*E*_22_*^t^*	Young’s tensile modulus in transverse direction
*E*_22_*^c^*	Young’s compressive modulus in transverse direction
FIA	Fédération Internationale de l’Automobile
*G*	Shear modulus
Soft	Softening reduction factor
Erods	Maximum effective strain parameter
FS	Failure Surface
TSize	Time step size parameter for automatic element deletion
SAE	SAE International
SLIM	Factors to determine the minimum stress limit after failure
